# Revisiting MMPBSA by Adoption of MC-Based Surface Area/Volume, ANI-ML Potentials, and Two-Valued Interior Dielectric Constant

**DOI:** 10.1021/acs.jpcb.3c00834

**Published:** 2023-05-12

**Authors:** Ebru Akkus, Omer Tayfuroglu, Muslum Yildiz, Abdulkadir Kocak

**Affiliations:** † Department of Chemistry, Gebze Technical University, 41400 Kocaeli, Turkey; ‡ Department of Molecular Biology and Genetics, Gebze Technical University, 41400 Kocaeli, Turkey

## Abstract

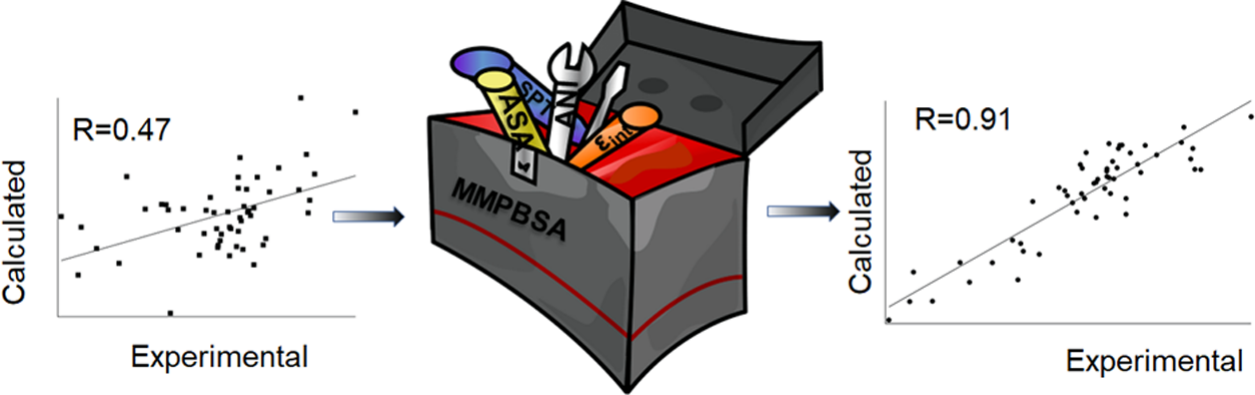

Here, we report the accuracy improvements of molecular mechanics Poisson–Boltzmann surface area (MMPBSA) calculations by adoption of ANI-ML potentials in replacement of MM terms, the use of solvent-accessible surface area (SASA) and volume (SAV) values from the Monte Carlo sampling of the probe, and introducing two different interior dielectric constants for electrostatic interactions of protein–ligand (P–L) and polar solvation term in the MMPBSA calculations. Our results show that the Pearson correlation coefficients of MMPBSA-calculated values with respect to experimental binding free energies can be drastically improved from 0.48 to 0.90 by adoption of ANI-ML potentials in replacement of MM energy terms in the equation, referred to as ANI-PBSA. Moreover, we show that the SASA/SAV-combined equation in the scaled particle theory (SPT) can be a better choice to model nonpolar solvation term, reaching nearly the same accuracy by ANI-PBSA calculations. Finally, we introduce two different values of interior dielectric constants, which could be an alternative strategy between the single and variable constant definitions.

## Introduction

Accurate and fast prediction of free-energy changes is a substantial research field in computational chemistry.^1^ All chemical and biomolecular events are governed by the foundation of thermodynamic processes.^2^ Experimental techniques can become too costly, time consuming, and not informative about the dynamic properties.^3−7^ In particular, in biomolecular processes, binding free energy of a ligand to a macromolecule has been a great deal in drug discovery. Computation of binding free energies (BFEs) and screening large libraries of potential drug candidates based on BFEs have been a routine process in rational drug design studies.^8,9^ However, computational studies require the most realistic modeling of the system. Due to the large size such that the biomacromolecule–small ligand complex is surrounded by explicit water molecules, the system cannot be simply simulated at the quantum mechanical (QM) level. At the current computational power, most parts of these systems, if not all, must be defined at the molecular mechanics (MM) level.^10^

Molecular dynamics (MD) simulations have been widely used for BFE calculations.^2^ The methods to calculate the BFEs using MD simulations can be grouped into two classes. First, BFEs are predicted by using only bound and unbound states of the system so called end-state methods. Second, the system is evolved from the bound to unbound state by physical or alchemical pathway via (ideally) infinitesimal changes of the energy function.^11^ Although the pathway methods are more rigorous, they do not perform well in the trade-off between accuracy and computational cost.^1^ On the contrary, the end-state methods, although more approximate methods, have been widely used in drug discovery.^12−16^ The most common end-state methods are linear interaction energy (LIE),^17^ molecular mechanics Poisson–Boltzmann surface area (MMPBSA), and generalized Born surface area (MMGBSA).^18−20^ LIE uses averaged ensembles of interaction energies between the ligand and surroundings in both bound and unbound states, and BFE is predicted by empirical coefficients produced by fitting to experimental data.

MMP(G)BSA methods find BFE by composing contributions from gas-phase binding energy (i.e., MM energy term), solvation, and entropy. The implicit solvation term is also assembled by the contribution of polar (PB or GB)^21^ and nonpolar solvation based on surface area (SASA) or volume (SAV) energy terms.^22^ These contributions to BFE can be collected by following either a separate trajectory protocol or single-trajectory protocol.

In the separate trajectory protocol (3A-MMP(G)BSA), independent MD simulations are performed for L in water (LS), apo-protein in water (PS), and protein–ligand complex in water (PLS). This protocol is a more realistic approach to the experimental process since the conformational sampling of apo-protein and free ligand may differ from that of the complex. However, running separate MD simulations may bring additional errors and noise due to large internal energy fluctuations at different conformations. In most cases, these errors cannot be fixed by longer MD simulations. Indeed, studies have shown that longer MD simulations worsen the BFE prediction of the MMP(G)BSA calculations. On the other hand, these errors can be minimized by increasing the number of replicas in simulations at the end points. Su et al. have shown that 4 ns of simulation time is sufficient along with 30–50 replica simulations.^23^

In the single-trajectory protocol (MMP(G)BSA), only a single MD simulation of PLS is performed, and the individual components of P and L are extracted from the same trajectory. Although this protocol suffers from the lack of conformational sampling of apo-protein and free ligand, the error due to the fluctuation of energy terms is much smaller since it is canceled due to the use of the same MD frames for each component. In addition, the computational cost is much less than the separate trajectory protocol. Therefore, the single-trajectory protocol is more widespread in the field.

There have been several attempts to improve the accuracy of MMP(G)BSA in the literature.^10,24−26^ Much progress has been made by the implementation of several ideas such as correcting MM terms with accurate QM calculations as in the case of linear scaling DFT^27^ or semiempirical quantum mechanical methods,^28^ correcting entropic term in the equation,^29^ and using variable dielectric constants in the solvation term.^30−32^ Wang et al. have recently reviewed these studies in detail.^1^ Most of the studies have been focused on correcting PB/GB and SA terms in the MMP(G)BSA energy formalism. Since the efficiency and accuracy of these methods are system-dependent, there is no consensus on the coefficients used to convert these terms in the energy equation. In Amber22 software, there are six regimes to calculate PBSA and regimes for GBSA contributions.

In the original MMPBSA calculation using SAV for the nonpolar solvation term, the volume is calculated by the integration of SASA,^21,33^ in which a spherical probe samples the accessible surface of the protein–ligand complex. Recently, Ongari et al.^34^ have introduced the use of accessible surface area (ASA), probe occupiable accessible volume (POAV), and probe-centered accessible volume (PCAV) for the internal voids of microporous materials available in Zeo++ software.^35^ In these strategies, the area and volume of cavities (or voids) are determined by Monte Carlo simulations of the spherical probe rather than the integration of SASA. Ideally, ASA in Zeo++ should correspond to SASA in AMBER. Similarly, PCAV corresponds to SAV in protein–ligand complexes except for the calculation method. POAV differs from PCAV by including the additional pore volume due to the probe radius, which is not considered in PCAV (or SAV).

Here, we attempt to improve the BFE prediction by thoroughly assessing the performance of MMP(G)BSA from four different perspectives. First, conventional MMPBSA is analyzed using different regimes covering SASA-only, SAV-only, and SPT models in nonpolar solvation contribution part of the binding free energy. Second, the applicability of using POAV/PCAV rather than SAV in the MMP(G)BSA has been tested. Next, ANI-ML potentials are adopted as a replacement of MM terms in the implicit solvent approach so called ANI_PBSA to predict BFEs with higher accuracy. In our previous work, we have introduced ANI_LIE method, which uses ANI-2x machine-learned (ML) potentials in the LIE formalism and showed significant improvements in BFE predictions over the classical LIE approach. Finally, the use of two different dielectric constants in the same calculation has been tested.

## Theory

### MMP(G)BSA

In the single-trajectory MMPBSA or MMGBSA, the free-energy change for binding of a ligand (L) to a protein (P) to form a complex (PL) can be expressed as
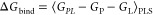
1where ⟨⟩_PLS_ shows the averaged ensemble from the simulation carried in protein–ligand–solvent (PLS) system. Here, each individual component (PL, P, and L) is extracted from each of MD frames. The binding free energy can be decomposed into^20^
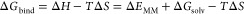
2in which
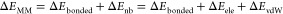
3where Δ*E*_MM_, Δ*G*_solv_, and −*T*Δ*S* are the changes in the gas-phase MM energy, solvation free energy, and conformational entropy changes upon ligand binding. Δ*E*_MM_ includes the energy from Δ*E*_bonded_-bonded terms (bond, angle, dihedral) and Δ*E*_nb_ nonbonding terms as Δ*E*_ele_ electrostatic and Δ*E*_vdW_ van der Waals interactions. For the noncovalent interaction between the protein and ligand, Δ*E*_bonded_ is basically zero and thus
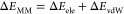
4

The solvation term is also decomposed as
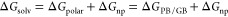
5where Δ*G*_PB/GB_ is the polar (electrostatic) contribution to the solvation free energy calculated by PB or GB model. Ideally, interior (solute) and exterior (solvent) dielectric constants are set to ε_int_ = 1 and ε_ext_ = 80, respectively.^36^ Previous studies have shown that the dielectric constant of the solute (interior dielectric constant) should be larger than 1 for highly charged solutes and thus need to be adjusted for better performance.^13,37−43^ Δ*G*_np_ is the nonpolar contribution between the solute and continuum solvent, which has been calculated by either solvent-accessible surface area (SASA) or solvent-accessible volume (SAV) enclosed by SASA.

#### One-Term (SA-Only) Model for Δ*G*_np_

The nonpolar solvation term is expressed by an empirical function of SASA or SAV in this model^44,45^
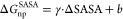
6
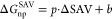
7where γ, *p*, and *b* are the surface tension constant, solvent pressure constant, and correction terms, respectively (γ = 0.00542 kcal·mol^–1^·Å^–2^ and *b* = 0.92 kcal/mol; γ = 0.005 kcal·mol^–1^·Å^–2^ and *b* = 0; or γ = 0.0072 kcal·mol^–1^·Å^–2^ and *b* = 0 in the AMBER package). The use of both SASA and SAV, which is called scaled particle theory (SPT),^45,46^ has also been tested and showed better results
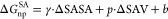
8

We note that the herein-described SPT model, just like the other one-term models, corresponds to repulsive terms only, Δ*G*_rep_ (i.e., ignores the attraction terms), in two-term model explained below.

It has been shown that for small spherical cavities, Δ*G*_rep_ is more correlated with the cavity volume for the cavity radius below 10 Å, whereas for larger cavity radius, it is more correlated with the cavity surface.^47−51^ The SPT model rather than SASA-only and SAV-only models could be more beneficial to reflect this crossover since it includes both terms in the equation.

#### Two-Term (Cavity-Dispersion) Model for Δ*G*_np_

The true nature of the nonpolar solvation term comes from repulsive and attractive components between solute and explicit solvents^44^
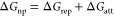
9where Δ*G*_rep_ is the solvation free-energy contribution from solute–solvent repulsive interactions and the formation of solute cavity. Δ*G*_att_ is the solvent–solute attractive nonpolar interaction, and it also includes the solvent–solvent reorganization component. Therefore, in the two-term model, nonpolar energy is further divided into cavity and dispersion contributions^44^
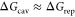
10
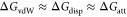
11
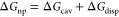
12where Δ*G*_cav_ is the cavity formation free energy corresponding to repulsive terms and Δ*G*_disp_ is the dispersion free energy corresponding to attractive nonpolar interactions between the solute and solvents. For the attractive term or dispersion contributions, several schemes have been tested such as using the attractive part of Lennard-Jones 6/12, Weeks–Chandler–Andersen (WCA), and σ-schemes.^44^ For the repulsive part, SASA or SAV has been used in the same formula of the one-term approach. So
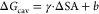
13
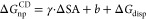
14

Similarly, the accessible volume instead of area can be used in these equations. The dispersion term, Δ*G*_disp_, can also be calculated by solvent-accessible volume (or surface) integration (the scaling factors, γ = 0.0378 kcal·mol^–1^·Å^–2^ and *b* = −0.5692 kcal/mol in the AMBER package). Although it is the integration of the area, it is still a direct function of solvent-accessible volume and can be approximated to
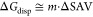
15

Implementing this approximation in the two-term formula, the equation becomes similar to one-term approach of SASA–SAV model (i.e., SPT theory) or SAV-only model with new coefficients.

If area as the repulsive (cavity) term is used
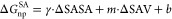
16

If the volume is used
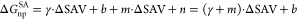
17

If both area and volume are used
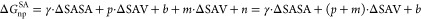
18

It is clear that when the dispersion energy term in the two-term model is linear to accessible volume, one-term model is reached in either case. The only change will be in the weight factors of SASA and SAV terms.

Recently, Zhang et al.^52−54^ have proposed a new approach for energy estimator, PBSA_E, which is defined as
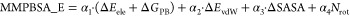
19where γ = 0.0072 kcal mol^–1^ Å^–2^ and α_1_, α_2_, α_3_, and α_4_ coefficients are 0.03037, 0.07791, 1.2193, and 0.1854, respectively. Here, *N*_rot_ was introduced as rotatable bonds to account for entropic contribution. This formula can be extended to entirely empirical parameters of all energy components and can be even represented by
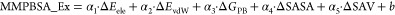
20

### ANI Replacement with MM Terms

Previously, we have shown that ANI-ML potentials can be utilized in the linear interaction energy (LIE) formalism by replacing ANI with the MM term.^55^ Herein, we borrow the same change in the MM/PBSA formalism so as to test the ANI_PBSA approach. The updated formula for the free energy of binding would be
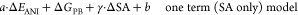
21
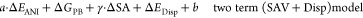
22
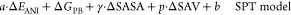
23
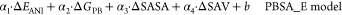
24In order to calculate the nonpolar contribution to the solvation free energy, we have assessed the use of ASA instead of SASA, PCAV, or POAV instead of SAV in all of the equations. Table 1 shows different regimes and formulas used to calculate MMPBSA throughout the manuscript.

**Table 1:** Different Regimes and Formulas Used to Calculate MMPBSA throughout the Manuscript

method	regime	nonpolar term	terms	table no.	equations involved
MMPBSA	one-term	SASA/ASA		2, 5	4, 5, 6
MMPBSA	one-term	SAV/PCAV/POAV		2, 5	4, 5, 7
MMPBSA	two-term	SAV/PCAV/POAV + Disp		3, 6	4, 5, 14
MMPBSA	SPT	SASA/ASA + SAV/PCAV/POAV		3, 6	4,5, 8
MMPBSA_E	one-term	SASA/ASA		4	19
MMPBSA_E (extended)	SPT	SASA/ASA + SAV/PCAV/POAV		4	20
ANI_PBSA	one-term	SA		6	21
ANI_PBSA	two-term	SAV + Disp		6	22
ANI_PBSA	SPT	SASA/ASA + SAV/PCAV/POAV		6	23
ANI_PBSAe	SPT	SASA/ASA + SAV/PCAV/POAV		6	24

## Computational Methods

### System Preparation

For the binding studies, the crystal structures of noncovalent ligands complexed with JNK-1, HIV-1, SARS-CoV, MERS-CoV, SARS-CoV-2 (we will refer to these five proteins as three protein families and use “data set A” throughout the manuscript), urokinase, HSP82, HSP90, endothiapepsin, AR, b-lactamase, Pim kinase, BRD4, endothiapepsin, FXIa, and PDE10A (we will use “data set B” for these 11 proteins), a total of 79 protein–ligand complex (16 different protein families) with known IC50/Kd/Ki values (Table S1), were retrieved from Protein Data Bank (PDB) except for c-Jun N-terminal kinases (JNK-1), which were retrieved from the work by Khalak et al.^56^ Using Gaussian 16 software, the model ligands were first optimized at the B3LYP/6-31G* and ESP charges were generated at the HF/6-31G* level. Using the Antechamber module in AmberTools 2021, RESP charges and GAFF force field atom types were generated. Amber2gmx module from AmberTools 2021^57^ was used to convert amber-type input files to Gromacs. All of the ligands in the current study were selected to be neutral with different polarities calculated by the SwissAdme^58^ web server (Table S2).

### MD Simulations

The molecular dynamics simulations were carried out using the Gromacs 2018+ software package^59,60^ with all-atom model of Amber ff99SB-ILDN^61−63^ force field implemented in Gromacs. The protein–ligand complex was placed in the center of a dodecahedron box. Each system was solvated in the TIP3P model-type water^64^ with a cell margin distance of 10 Å for each dimension. The protein–ligand systems with ∼70,000 atoms were neutralized in 0.15 M NaCl. Classical harmonic motions of all bonds were constrained to their equilibrium values with the LINCS algorithm.

Energy minimization was carried out to a maximum of 100 kJ mol^–1^ nm^–1^ force using the Verlet cutoff scheme. For both long-range electrostatic and van der Waals interactions, a cutoff length of 12 Å was used with the particle mesh Ewald method (PME) (4th-order interpolation). The neighbor list update frequency was set to 20 ps^–1^. As with our earlier studies,^65−67^ two-step energy minimization and equilibration schemes were used. Each minimization step consisted of up to a 50,000 cycle of steepest descent and a subsequent 50,000 cycles of l-bfgs integrators.

After minimization, each system was equilibrated within three steps using Langevin dynamics. The first step consisted of 1 ns of NVT ensemble. The protein–ligand and rest of the system were defined as two temperature groups at 310 K. The next steps consisted of NPT ensembles in which the systems were equilibrated to the 1 atm pressure by Berendsen for 200 ps and followed by the Parrinello–Rahman isotropic pressure coupling for 1 ns to a reference pressure of 1 atm. When systems reached equilibrium, an MD simulation of 10 ns was carried out at the NPT ensemble.

### PBSA Calculations

MMPBSA calculations were performed using the trajectories of Gromacs MD simulations via gmx_MMPBSA package.^68,69^ Our custom scripts were used to combine ANI and PBSA calculations as ANI_PBSA.

Statistical analyses were performed by using Igor Pro and custom python scripts for predictive index (PI) as outlined in ref (70), Pearson R, Spearman R, MUE, MUEtr, and MUEsc.^13^

## Results and Discussion

### MMPBSA by SASA and/or SAV

Single-trajectory approach MMP(G)BSA calculations can be carried out using several schemes as outlined in the Theory section. The most popular methods, which are also embedded in Amber22 software, are SASA-only and SAV + Disp models. In addition, the SAV-only model can be modulated, but it mostly mimics the results of SASA-only model. Although SASA is in area unit with numeric values ranging from ∼10,000 to 15,000 Å^2^ and SAV is in the volume unit ranging from ∼40,000 to 80,000 Å^3^ for protein–ligand (PL) complexes, the difference values (PL–P–L) on both SASA and SAV are very similar in the range of −500 to −1000 Å^2^ or Å^3^ (Supporting Information), and thus the coefficient values of γ and *p* in eqs 1 and 2 are very similar; thus, they can be simply represented with the same coefficient. Therefore, SASA-only model and SAV-only model yield almost identical results in reproducing experimental relative binding free energies (RBFE).

**Figure 1:**
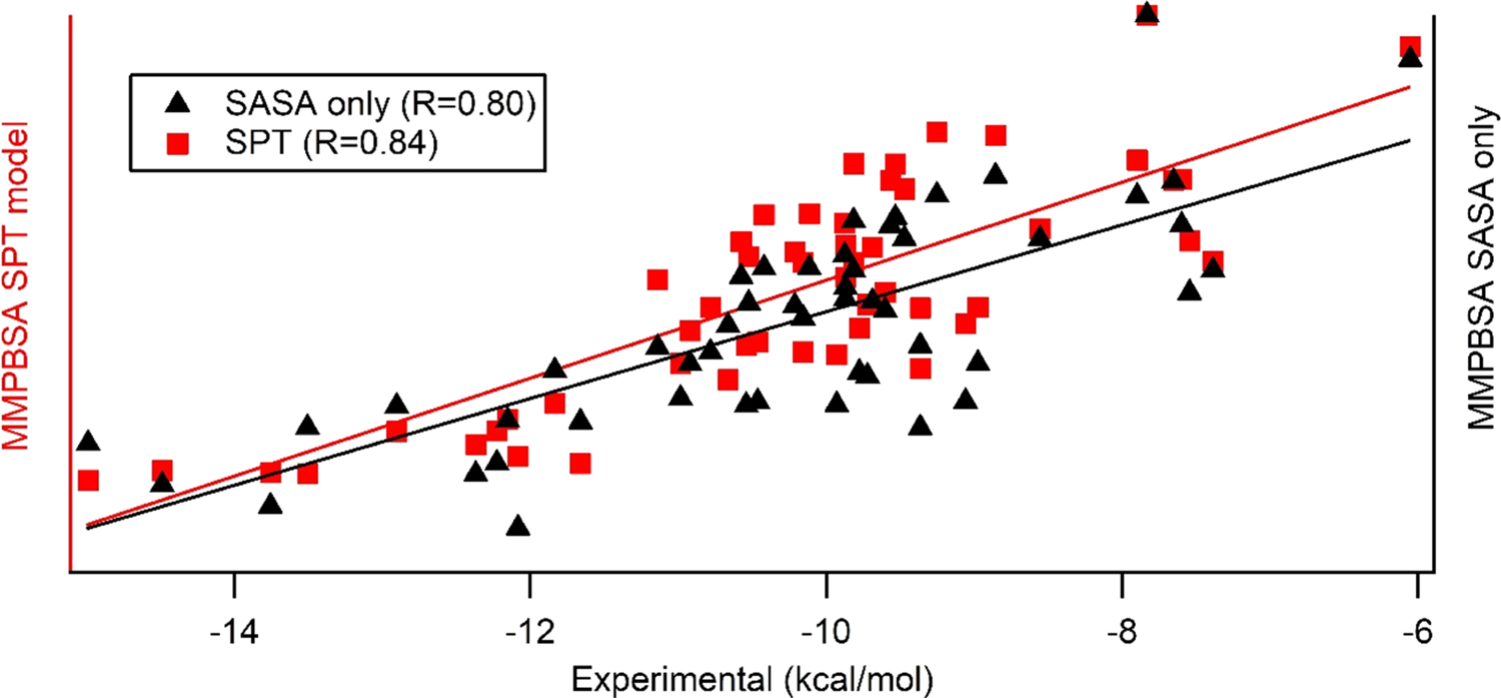
Experimental and calculated binding free energies by MMPBSA using SASA only/SPT regimes for nonpolar contribution where ε_int_ = 2.1 for electrostatic and polar solvation terms.

We have first tested the performance of predefined coefficients using SASA (or SAV)-only model (Table 2). Setting the interior dielectric constant to 1 (ε_int_ = 1), we could only get Pearson’s correlation coefficient, *R* = 0.42–0.47. However, when ε_int_ = 2.1, we observed a drastic increase in the correlation coefficient to *R* = 0.78–0.79. We have also tested different ε_int_ values (up to 10) and obtained no further significant improvement (Supporting Information). In addition to the single-term nonpolar model, we have also tested the two-term approach in which SASA (or SAV) is combined with dispersion (Disp) using predefined values of γ = 0.0378 and *b* = −0.5692. Unfortunately, this yielded very poor correlation (even reverse correlation) to the experimental values with ε_int_ = 1. By increasing ε_int_ (to 10), we could produce *R* = 0.50 correlation. More of ε_int_ will be discussed later in the manuscript. Instead of using predefined coefficients, we have also fitted the SASA (or SAV) in both one- and two-term models using the experimental BFE of the 54 PL complexes studied here. For the one-term model, we improved the correlation by 5% (using γ = 0.08) with ε_int_ = 1 or 2.1. This correlation could not be further improved by changing ε_int_. In the SPT model, which is not a default model in Amber22, we produced a slightly better fit to the experimental free energies (*R* = 0.84). Figure 1 shows correlation between experimental binding free-energy values of PL complexes and SASA only/SPT regimes by setting ε_int_ = 2.1. Interestingly, the success of this model was not affected by different ε_int_ values. Table 2 shows the summary of Pearson coefficients produced by original and our fit parameters.

**Table 2:** MMPBSA Calculated by One-Term, Two-Term, and SPT Approaches for Nonpolar Contribution to Solvation Free Energy on Data Set A, in Which Fitting Was Performed (For the Test Set of Data Set B, Refer to Table S2)^a^

	SASA_only			SAV_only			SAV + Disp		SPT
ε_int_ = 1	GB1 = PB4, γ = 0.0072	GB2 = PB5, γ = 0.005	our, γ = 0.0836	GB1 = PB4, γ = 0.0072	GB2 = PB5, γ = 0.005	our, γ = 0.0785	PB1γ = 0.0378, *b* = −0.5692	our, γ = 0.1481	ourγ = 0.1394, *p* = 0.0163
PI	0.42	0.36	0.86	0.43	0.36	0.85	–0.51	0.83	0.87
Pearson R	0.47	0.42	0.84	0.47	0.42	0.82	–0.54	0.80	0.84
Spearman R	0.39	0.34	0.80	0.40	0.33	0.77	–0.45	0.76	0.80
MUE	24.52	22.31	101.35	24.46	22.26	95.51	10.70	99.92	101.86
MUEtr	3.79	3.84	13.34	3.85	3.87	13.00	7.40	13.24	13.32
MUEsc	2.93	3.44	1.04	2.93	3.43	1.10	2.61	1.20	1.03

ε_int_ = 2.1			γ = 0.0582			γ = 0.0538		γ = 0.1234	γ = 0.0933, *p* = 0.0343
PI	0.83	0.83	0.85	0.82	0.83	0.84	–0.13	0.82	0.86
Pearson R	0.79	0.79	0.84	0.79	0.78	0.83	–0.11	0.80	0.84
Spearman R	0.76	0.75	0.78	0.74	0.75	0.76	–0.09	0.75	0.78
MUE	39.20	36.99	90.46	39.14	36.95	85.62	5.39	90.03	91.58
MUEtr	5.31	5.00	13.26	5.42	5.08	12.92	3.88	13.26	13.17
MUEsc	1.22	1.25	1.07	1.26	1.28	1.11	15.11	1.23	1.06

a PI: predictive index; MUE: mean unsigned error; MUEtr: mean unsigned error after subtraction of the average signed error; and MUEsc: MUE rescaled by the slope and intercept of the predicted vs experimental results.

In order to test the applicability of the method to different protein–ligand complexes, we have used the same coefficients for a test set of 25 new protein–ligand complexes from the CASF-2016 data set. The same conclusion of the SPT model’s superiority over the other model was still observed (Table S2).

**Figure 2:**
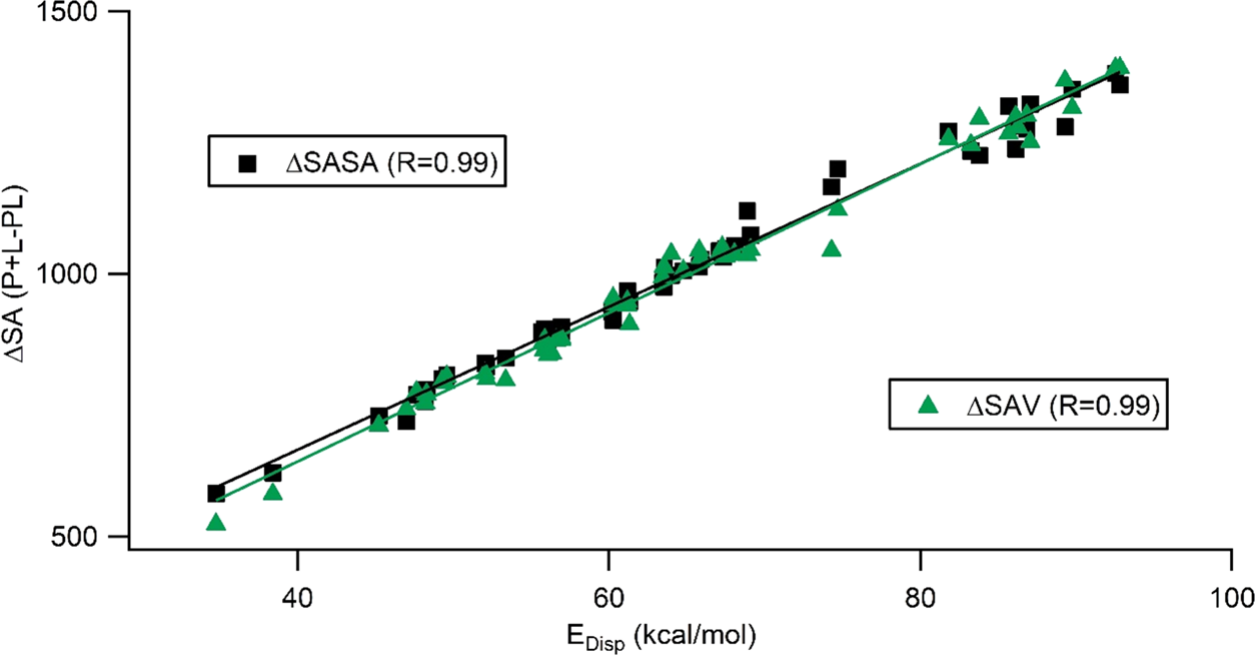
Relationship between SASA (or SAV enclosed by SASA) and *E*_disp_ (calculated by the integration of SASA). The values are of averages of PL–P–L differences over 100 MD snapshots.

As discussed in the Theory section, when the dispersion energies are a linear function of SASA, the SAV + Disp model should converge to the same equation as the SPT model (with new weight factors). Figure 2 shows the correlation between dispersion energy and SASA (or SAV enclosed by SASA). Therefore, using SASA + SAV terms (SPT model) instead of SAV + Disp (two-term model) should produce very similar results. We find that the SPT model has slightly better results than the SAV + Disp model since the coefficient for Disp is set to 1 (i.e., not a fitting parameter) in the case of SAV + Disp, whereas it is also fitted in the SPT model.

When all energy terms are defined with empirical parameters as in the MMPBSA_E formalism in eq 19 using original values of coefficients, we observed the Pearson correlation of 0.84, which is almost the same as the SPT model. However, when we used our fitting parameters, we could slightly improve the correlation to 0.85–0.86. In this model, electrostatic MM energy and polar solvation energy difference (Δ*E*_ele_ and Δ*G*_PB_) are scaled with the same scaling factor. The logical idea is that both terms are electrostatic and thus a single coefficient, which involves the interior dielectric constant, could be used to represent all of the electrostatic terms in the system. Using the extended version of MMPBSA_Ex in eq 20, the correlation can be improved up to 0.91. Table 3 shows Pearson correlation coefficients using all of these different regimes. Similar improvement was also observed for the data set of 25 complexes.

**Table 3:** MMPBSA_E by SASA Only and MMPBSA_Ex (Extended) by SPT Models for Nonpolar Contribution to Solvation Free Energy^a^

	ε_int_ = 1				ε_int_ = 2.1			
	MMPBSA_E by SASA only			MMPBSA_Ex by SPT	MMPBSA_E by SASA only			MMPBSA_Ex by SPT
	original		our fit	our fit	original		our fit	our fit
	α_1_ = 0.0303		α_1_ = 0.453	α_1_ = 0.969	α_1_ = 0.0303		α_1_ = 0.881	α_1_ = 2.002
	α_2_ = 0.0779		α_2_ = 0.575	α_2_ = 0.389	α_2_ = 0.0779		α_2_ = 0.557	α_2_ = 0.367
	α_3_ = 0.0072		α_3_ = 0.050	α_3_ = 0.461	α_3_ = 0.0072		α_3_ = 0.050	α_3_ = 0.932
				α_4_ = 0.009				α_4_ = 0.008
				α_5_ = −0.036				α_5_ = −0.004
	data set	data set	data set	data set	data set	data set	data set	data set
	A	B	A	A	A	B	A	A
PI	0.85	0.90	0.86	0.90	0.84	0.90	0.86	0.90
Pearson R	0.84	0.88	0.85	0.91	0.84	0.87	0.85	0.91
Spearman R	0.77	0.87	0.79	0.86	0.77	0.87	0.79	0.86
MUE	0.98	1.81	90.46	62.38	1.16	1.63	91.13	62.81
MUEtr	0.94	1.11	12.98	12.26	0.99	1.11	12.98	12.27
MUEsc	1.08	1.24	1.03	0.77	1.10	1.26	1.02	0.77
MUEsc	0.85	0.90	0.86	0.90	0.84	0.90	0.86	0.90

a PI: predictive index; MUE: mean unsigned error; MUEtr: mean unsigned error after subtraction of average signed error; and MUEsc: MUE rescaled by the slope and intercept of the predicted vs experimental results.

### ASA/PCAV(POAV) in Replacement of SASA/SAV

**Figure 3:**
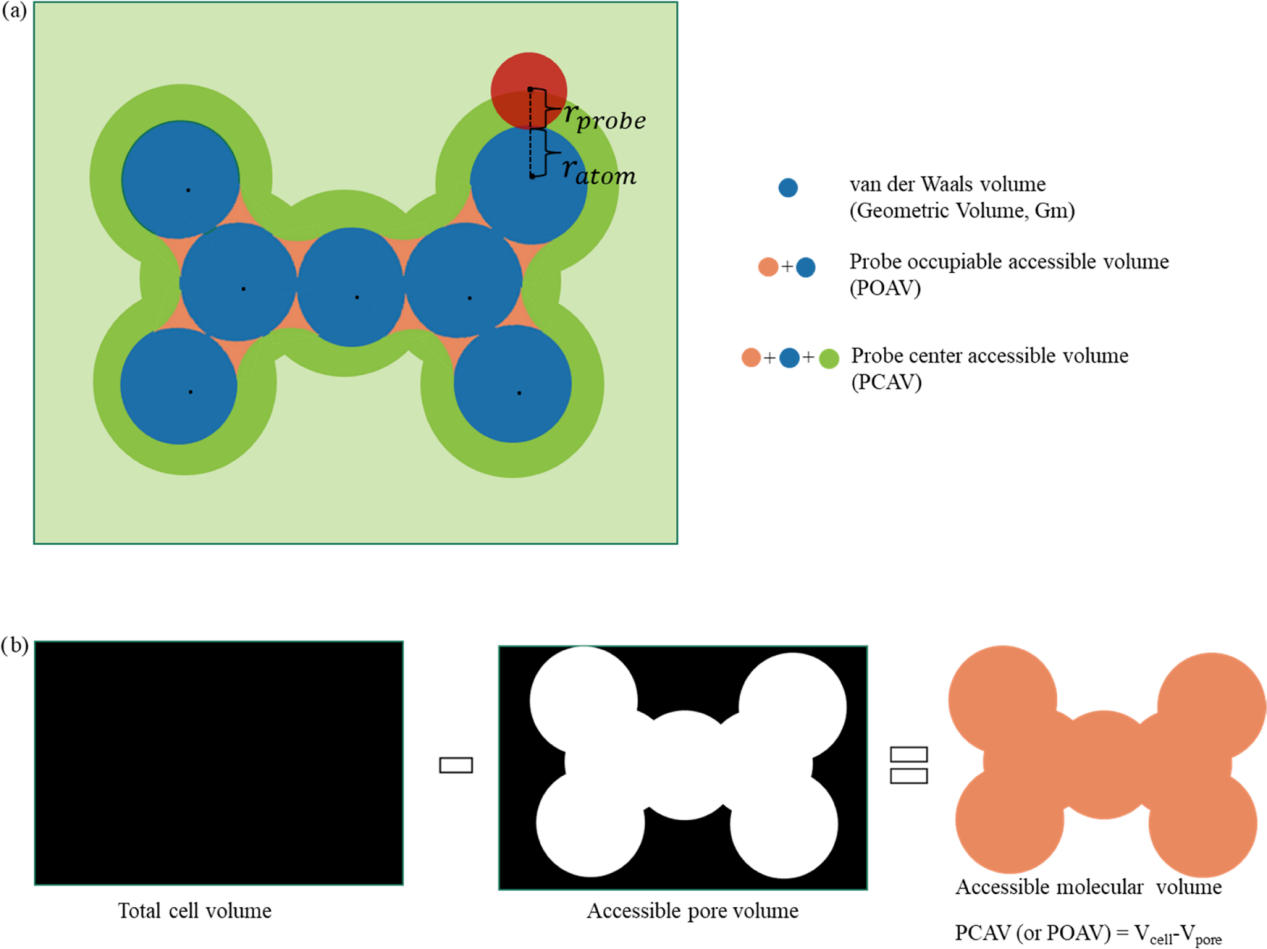
Illustration of pore volume produced by Zeo++ showing (a) covered regions in PCAV and POAV definitions and (b) how we calculated the molecular surface from pore volume.

In addition to the results of the superiority of the SPT model over the other tested schemes, we also investigated the area and volume calculations using a different approach. In Zeo++, the accessible surface area (ASA), probe-centered accessible volume (PCAV), and probe occupiable accessible volume (POAV) could be utilized for the effect of area/volume on the success of binding free estimation in MMPBSA calculations. Ideally, ASA corresponds to SASA and PCAV corresponds to SAV. The only difference is that Zeo++ uses Monte Carlo sampling of the probe in the calculation of void of the bulk structure. Since we are interested in molecular surface rather than pore volume, we have subtracted Zeo++-calculated pore volumes from the total volume of the cell (Figure 3). On the other hand, POAV eliminates the volume, which is overcalculated due to the probe radius.

We first compared the values of ASA generated by Zeo++ instead of SASA from the Amber software. They are almost identical, and as expected there is no improvement in the MMPBSA calculations when SASA is replaced by ASA. On the other hand, PCAV generated by Zeo++ and SAV from the Amber software produced slightly different results (Table 4). This was the case for both data sets A and B (Table S3). When the MMPBSA equations are modified to include ASA/PCAV rather than SASA/SAV in the SPT model, the *R* values are slightly improved from 0.84 to 0.87, whereas the use of ASA/POAV has yielded very similar results with the original SPT model (i.e., SASA/SAV). Similarly, MMPBSA_Ex is also improved by ∼3% (to *R* = 0.91) by using ASA/PCAV or ASA/POAV instead of SASA/SAV.

**Table 4:** MMPBSA Calculated by Zeo++ Produced SA Terms for Nonpolar Contribution to Solvation Free Energy^a^

	one-term			two-term		SPT	
ε_int_ = 1	ASA only	PCAV only	POAV only	PCAV + Disp	POAV + Disp	ASA + PCAV	ASA + POAV
	γ = 0.08525	γ = 0.06748	γ = −0.1150	γ = 0.1327	γ = −0.1907	γ = 0.1151,p = −0.0326	γ = 0.0797,p = −0.0299
PI	0.87	0.74	0.55	0.45	–0.36	0.88	0.87
Pearson R	0.85	0.70	0.53	0.45	–0.31	0.86	0.85
Spearman R	0.81	0.66	0.47	0.38	–0.34	0.83	0.80
MUE	101.87	70.75	24.77	57.44	35.28	105.67	98.29
MUEtr	13.36	11.38	6.42	13.03	13.19	13.49	13.25
MUEsc	1.02	1.63	2.28	3.35	4.85	0.94	0.97

ε_int_ = 2.1	γ = 0.0594	γ = 0.0414	γ = −0.0925	γ = 0.1067	γ = −0.1682	γ = 0.1052, *p* = −0.05	γ = 0.0527, *p* = −0.0362
PI	0.86	0.80	0.73	0.52	–0.23	0.88	0.85
Pearson R	0.84	0.75	0.77	0.50	–0.13	0.87	0.85
Spearman R	0.78	0.71	0.64	0.45	–0.22	0.82	0.77
MUE	90.92	64.82	37.98	51.50	22.38	96.73	86.58
MUEtr	13.31	11.17	7.57	12.28	8.91	13.12	13.06
MUEsc	1.05	1.46	1.34	2.98	11.07	0.91	1.01

a PI: predictive index; MUE: mean unsigned error; MUEtr: mean unsigned error after subtraction of the average signed error; and MUEsc: MUE rescaled by the slope and intercept of the predicted vs experimental results.

### ANI in Replacement of MM Terms (ANI_PBSA)

As discussed in the Theory section, ANI has been tested using several schemes in MMPBSA calculations. Instead of MM energy terms so as to estimate the protein–ligand gas-phase binding free-energy contribution to the total binding free energy of solvated systems, single-point energies produced by ANI could be used (eqs 15–18) referred to as “ANI_PBSA”. Introducing ANI, we have observed a dramatic increase in the correlation plots in almost all of the different regimes (Tables 5, 6, and S4) of PBSA calculations with original parameters.

**Table 5:** ANI_PBSA Calculated by AMBER Produced SA Terms for Nonpolar Contribution to Solvation Free Energy^a^

	one-term				two-term		SPT	
	SASA only		SAV only		SAV + Disp		SASA + SAV	
ε_int_ = 1	*a* = 1.5508, γ = 0.0609		*a* = 1.6187, γ = 0.0511		*a* = 1.6497, γ = 0.1184		*a* = 1.7478, γ = 0.1112, *p* = −0.0636	
	data set	data set	data set	data set	data set	data set	data set	data set
	A	B	A	B	A	B	A	B
PI	0.82	0.69	0.81	0.67	0.79	0.66	0.80	0.69
Pearson R	0.79	0.68	0.78	0.68	0.76	0.66	0.80	0.67
Spearman R	0.74	0.64	0.73	0.63	0.69	0.63	0.73	0.63
MUE	76.05	50.70	69.17	45.87	72.87	48.82	73.28	49.74
MUEtr	13.91	20.66	13.54	22.15	13.70	23.77	13.99	18.83
MUEsc	1.17	2.57	1.24	2.56	1.34	2.71	1.16	2.64

ε_int_ = 2.1	*a* = 1.1425, γ = 0.0442		*a* = 1.2554, γ = 0.0316		*a* = 1.2863, γ = 0.098937		*a* = 1.3757, γ = 0.10362, *p* = 0.075252	
PI	0.84	0.83	0.83	0.85	0.82	0.81	0.85	0.89
Pearson R	0.85	0.81	0.84	0.81	0.82	0.79	0.86	0.85
Spearman R	0.76	0.80	0.75	0.83	0.72	0.79	0.77	0.86
MUE	67.19	47.54	60.09	42.80	63.78	45.74	213.96	156.01
MUEtr	13.44	16.32	13.07	17.02	13.30	18.32	38.04	46.32
MUEsc	0.98	1.71	1.02	1.76	1.12	1.93	0.98	1.34

a PI: predictive index; MUE: mean unsigned error; MUEtr: mean unsigned error after subtraction of the average signed error; and MUEsc: MUE rescaled by the slope and intercept of the predicted vs experimental results.

**Table 6:** ANI_PBSA Calculated by Zeo++ Produced SA Terms for Nonpolar Contribution to Solvation Free Energy (For the Data Set B, Refer to Table S4)^a^

	one-term			two-term		SPT	
	ASA only	PCAV only	POAV only	PCAV+EDisp	POAV+EDisp	ASA_PCAV	ASA_POAV

ε_int_ = 1	*a* = 1.4993, γ = 0.0662	*a* = 2.4414, γ = −0.0212	*a* = 2.1347, γ = −0.0498	*a* = 2.9684, γ = 0.0072	*a* = 2.922, γ = −0.07088	*a* = 1.6755, γ = 0.1139, *p* = −0.0669	*a* = 1.5568, γ = 0.0562, *p* = −0.0300
PI	0.83	0.79	0.78	0.70	0.70	0.86	0.81
Pearson R	0.80	0.77	0.78	0.69	0.72	0.84	0.80
Spearman R	0.75	0.70	0.70	0.60	0.60	0.81	0.73
MUE	77.77	43.63	47.96	28.26	24.76	81.15	72.74
MUEtr	13.93	13.41	13.92	15.04	15.05	14.22	13.87
MUEsc	1.15	1.29	1.31	1.67	1.51	1.02	1.16

ε_int_ = 2.1	*a* = 1.1029, γ = 0.0482	*a* = 1.8698, γ = −0.0232	*a* = 1.5612, γ = 0.0389	*a* = 2.3969, γ = 0.0052,	*a* = 2.3485, γ = −0.0599	*a* = 1.2596, γ = 0.0907, *p* = −0.0596	*a* = 1.1504, γ = 0.0399, *p* = −0.0248
PI	0.84	0.83	0.82	0.76	0.77	0.89	0.84
Pearson R	0.85	0.84	0.84	0.76	0.79	0.89	0.86
Spearman R	0.76	0.76	0.74	0.66	0.68	0.84	0.77
MUE	68.53	41.66	46.77	26.30	23.56	71.56	64.38
MUEtr	13.47	12.90	13.41	14.02	14.14	14.02	13.58
MUEsc	0.97	1.04	1.05	1.35	1.27	0.86	0.98

a PI: predictive index; MUE: mean unsigned error; MUEtr: mean unsigned error after subtraction of the average signed error; and MUEsc: MUE rescaled by the slope and intercept of the predicted vs experimental results.

In addition, ANI_PBSA calculations were compared to the MMPBSA calculations with our fit parameters. The correlation coefficient by ANI_PBSA calculations is either comparable to or even slightly better than MMPBSA calculations with our fit parameters when ε_int_ = 1 or 2.1. In particular, when ANI is implemented in the SPT formalism using either ASA + PCAV or SASA + SAV (i.e., eq 23), we produced the highest correlation as much as *R* = 0.89 and 0.90, respectively. This is almost a 5% improvement over MMPBSA using the same formalism (SPT).

**Figure 4:**
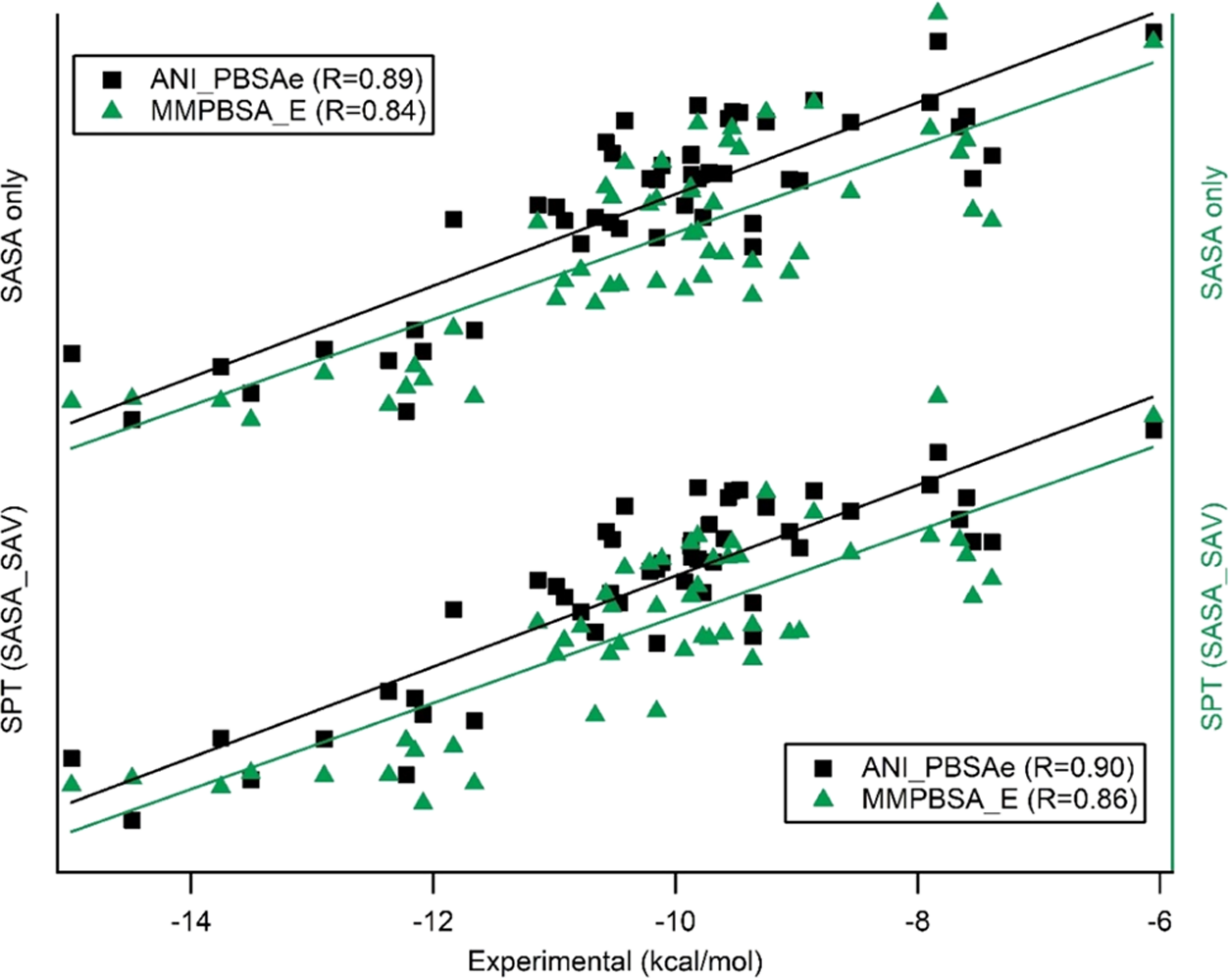
Comparison of MMPBSA_E and ANI_PBSAe by using SASA only and SPT regimes.

ANI has also been implemented in the extended form of PBSA_E formalism named “ANI_PBSAe”. In almost all of the different regimes, introducing ANI as a replacement for the MM term increases the correlation coefficient. Using SASA + SAV or ASA + PCAV in the SPT scheme, ANI_PBSAe yields 0.90 and 0.91, respectively (Table 7). The correlation coefficient was not affected by changing ε_int_, but the fitting parameter of the PB term in the equation is linearly dependent of ε_int_ as expected. Figure 4 shows the comparison of MMPBSA_E and ANI_PBSAe by using SASA only and SPT regimes, respectively. On the contrary to data set A, slightly lower correlations were observed for the data set of 25 complexes.

**Table 7:** ANI_PBSAe Calculated by SPT Model for Nonpolar Contribution to Solvation Free Energy^a^

	SASA + SAV		ASA + PCAV		ASA + POAV	
ε_int_ = 1	α_1_ = 0.7568, α_2_ = 0.4126, α_3_ = 0.0850, α_4_ = 0.0882		α_1_ = 0.7294, α_2_ = 0.2250, α_3_ = 0.0589, α_4_ = 0.0492		α_1_ = 0.5403, α_2_ = −0.3433, α_3_ = 0.0150, α_4_ = 0.0144	
	data set	data set	data set	data set	data set	data set
	A	B	A	B	A	B
PI	0.87	0.79	0.90	0.76	0.86	0.85
Pearson R	0.90	0.71	0.91	0.71	0.89	0.80
Spearman R	0.81	0.76	0.85	0.70	0.79	0.83
MUE	13.50	41.93	13.89	44.15	10.65	42.41
MUEtr	0.80	18.60	0.79	17.62	0.84	16.62
MUEsc	0.87	2.27	0.90	2.20	0.86	1.73

ε_int_ = 2.1	α_1_ = 0.7633, α_2_ = 0.8606, α_3_ = 0.0834, α_4_ = 0.0863		α_1_ = 0.7349, α_2_ = 0.4710, α_3_ = 0.0589, α_4_ = 0.0491		α_1_ = 0.5433, α_2_ = 0.7327, α_3_ = 0.0150, α_4_ = 0.0137	
PI	0.87	0.64	0.90	0.76	0.85	0.85
Pearson R	0.90	0.60	0.91	0.71	0.89	0.80
Spearman R	0.81	0.57	0.85	0.70	0.78	0.83
MUE	13.49	49.28	13.89	44.37	13.34	42.76
MUEtr	0.80	22.03	0.79	17.68	0.85	16.80
MUEsc	0.87	2.99	0.90	2.19	0.85	1.73

a PI: predictive index; MUE: mean unsigned error; MUEtr: mean unsigned error after subtraction of the average signed error; and MUEsc: MUE rescaled by the slope and intercept of the predicted vs experimental results.

### Two-Valued ε_int_ in MMPBSA/ANI-PBSA Calculations

In the original MMPBSA formalism, ε_int_, which corresponds to the solute dielectric constant, is set to 1. The use of a single solute dielectric constant for ligand–receptor complexes, which are not uniform dielectric media, is controversial and can cause significant errors.^13,32,37,43,71−74^ There have been numerous attempts to correct the MMPBSA calculations by adjusting ε_int._^13,33,75−78^ One of the most promising methods is to use a variable dielectric constant, which take different values on acidic, basic, and neutral amino acids in the protein.^32^ However, most calculations still use a single value of ε_int_, which is set to 2–4 for larger data sets of diverse proteins.^43,79,80^

Although the attempts were focused on correcting the solvation free-energy part of the MMPBSA calculations by adjusting the dielectric constant of the solute, it is appeared in two terms, and in both, it is inversely proportional to energy. The first is the Coulombic energy term of the MM part of P–L and the second is the polar solvation term of the PB/GB calculations. Thus, the total binding free energy is affected by these two altered values.

Our MMPBSA calculations using default parameters in Amber software yielded comparatively poorer results with ε_int_ = 1. When this value is increased to 2.1, we have observed a significant improvement in the correlation coefficient with greater MUEs. However, when further increased up to 10, negligible improvements were observed (Supporting Information)

**Figure 5:**
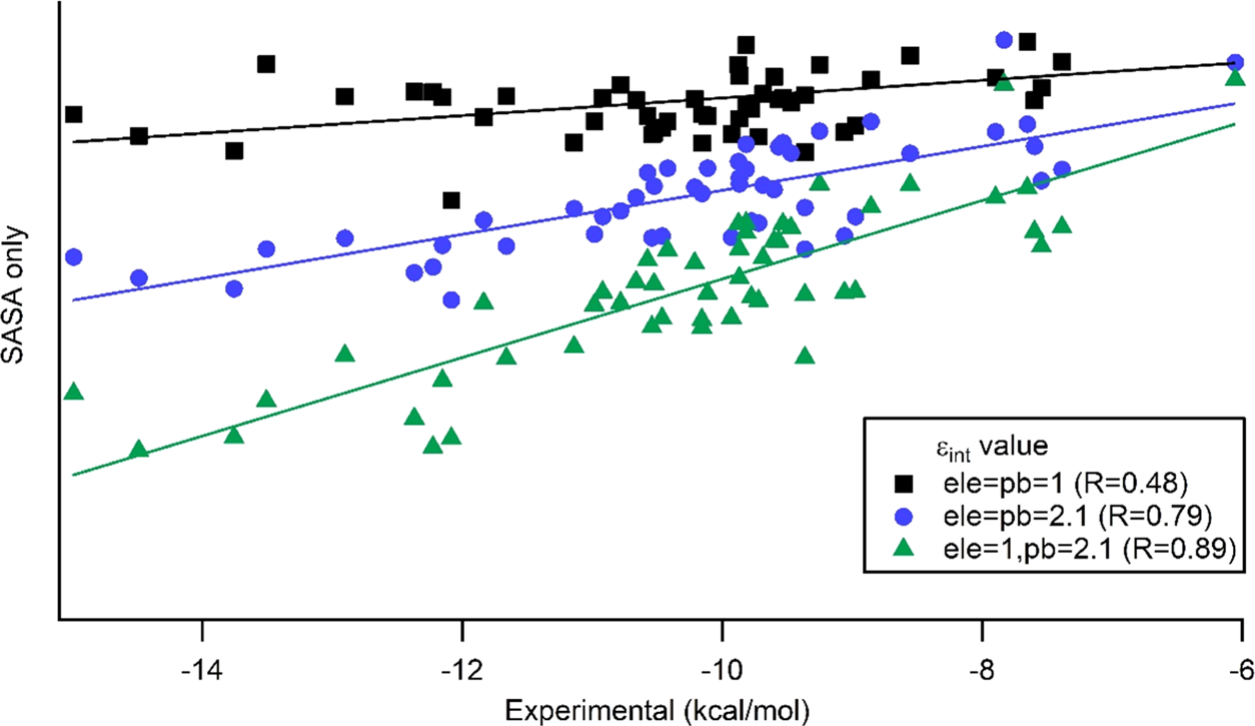
Comparison of the single-valued vs two-valued ε_int_ for Coulombic (P–L) and polar solvation terms, respectively.

Instead of using a single dielectric constant for both protein–ligand electrostatic term and polar solvation free-energy change upon complexation, we considered a different approach and defined two ε_int_ values (Figure 5), one for the Coulombic term of P–L and the other for the PB term of (PL–P–L).

The rationale behind this is that the neutral ligand is mostly buried in the binding pocket whose amino acids are mostly neutral. Therefore, the ε_int_ of the Coulombic term appearing in the MM term must not be drastically changed. On the other hand, amino acids that are surface-exposed have direct interaction with the solvent. Thus, ε_int_ in the PB term must be optimized to better estimate solvation energies. Using this idea, we defined divalent ε_int_ in which it is fixed at 1 for the P–L Coulombic term, whereas various values of ε_int_ were used for the polar solvation term (from 2 to 10). Our results showed an improvement of ∼10% in the correlation coefficient in all cases. Tables 8 and 9 show the comparison between single-valued and two-valued ε_int_ results.

**Table 8:** Bivalent Epsilon Calculated by MMPBSA Produced SA Terms for Nonpolar Contribution to Solvation Free Energy

	one-term				two-term		SPT	
ε_ele_ = 1, *pb* = 2.1	SASA only		SAV only		SAV + Disp		SASA + SAV	
	γ = 0.0360, *b* = 0		γ = 0.0319, *b* = 0		γ = 0.1015, *b* = 0		γ1 = 0.10301, *p* = −0.0655, *b* = 0	
	data set	data set	data set	data set	data set	data set	data set	data set
	A	B	A	B	A	B	A	B
PI	0.88	0.93	0.87	0.93	0.85	0.93	0.90	0.90
Pearson R	0.89	0.92	0.88	0.92	0.85	0.92	0.90	0.88
Spearman R	0.83	0.92	0.82	0.92	0.79	0.92	0.84	0.88
MUE	82.44	63.51	78.01	60.20	82.42	63.51	84.58	65.22
MUEtr	12.98	21.29	12.66	21.19	12.95	21.78	13.10	20.24
MUEsc	0.84	1.02	0.89	1.00	0.99	1.03	0.79	1.27

**Table 9:** Bivalent Epsilon Calculated by Zeo++ Produced SA Terms for Nonpolar Contribution to Solvation Free Energy

	one-term			two-term		SPT	
data set A							
ε_ele_ = 1, *pb* = 2.1	ASA only	PCAV only	POAV only	PCAV + EDisp	POAV + EDisp	ASA_PCAV	ASA_POAV
	γ = 0.0368, *b* = 0	γ = 0.0256, *b* = 0	γ = −0.0642, *b* = 0	γ = 0.0909, *b* = 0	γ = −0.1399, *b* = 0	γ1 = 0.0653, γ2 = 0.0311, *b* = 0	γ1 = 0.0310, γ2 = 0.0311, *b* = 0
PI	0.89	0.86	0.26	0.70	–0.74	0.90	0.87
Pearson R	0.89	0.86	0.21	0.70	–0.64	0.90	0.88
Spearman R	0.84	0.79	0.21	0.60	–0.66	0.85	0.81
MUE	82.76	66.57	8.63	53.26	129.61	86.38	52.36
MUEtr	13.04	11.34	6.94	11.48	28.87	13.42	8.72
MUEsc	0.83	0.95	7.51	1.73	2.03	0.81	0.91

data set B							
ε_ele_ = 1, *pb* = 2.1	ASA only	PCAV only	POAV only	PCAV + EDisp	POAV + EDisp	ASA_PCAV	ASA_POAV
	γ = 0.0368, *b* = 0	γ = 0.0256, *b* = 0	γ = −0.0642, *b* = 0	γ = 0.0909, *b* = 0	γ = −0.1399, *b* = 0	γ1 = 0.0653, γ2 = 0.0311, *b* = 0	γ1 = 0.0310, γ2 = 0.0311, *b* = 0
PI	0.93	0.94	0.10	0.93	0.09	0.86	0.88
Pearson R	0.91	0.92	0.07	0.88	–0.17	0.86	0.87
Spearman R	0.91	0.93	–0.02	0.93	0.04	0.83	0.85
MUE	62.99	52.85	14.19	45.79	15.77	63.88	60.87
MUEtr	20.58	19.71	10.02	19.31	16.21	19.71	18.58
MUEsc	1.07	1.03	35.77	1.25	12.48	1.37	1.35

Similarly, if the ligand is bound from the surface of the protein and the interaction is highly electrostatic, ε_int_, which defines the MM term between the protein and ligand, can still be optimized to greater values than 1. In this case, two-valued ε_int_ still becomes superior to the single-valued one. When the ligand binding pocket is solvent-exposed, the two-valued ε_int_ may produce the same results as single ε_int_ due to the similarity between the binding pocket and surface of the protein in terms of the electrostatic nature.

In addition to data sets A and B, we also validated two-valued ε_int_ approach by reproducing previously reported MMPBSA values on two different data sets. We have selected the works by Hou et al.^37^ and Pandey et al.,^81^ who have reported explicit decomposed energy terms for several protein–ligand complexes. Both authors reported data using specific values of dielectric constants. However, since their data had decomposed energy terms, we have easily reproduced electrostatic and PB terms by simply dividing different ε values in the range of 1–10. Therefore, we could successfully compare the performance of using either single-valued ε or two-valued one. Table 10 shows the correlation between recalculated free-energy values and experimental BFEs.

**Table 10:** Comparison of the Performance of Pearson Correlation Coefficients Using Single-Valued vs Two-Valued ε after Reproduction of Data from Refs (37) and (81)

# complex		single-valued *R*				two-valued *R*				# charged a.a.	ref
(ε_ele_, ε_pb_)		(1, 1)	(2, 2)	(4, 4)	best	(1, 2)	(1, 4)	(2, 4)	best		(37)
avidin	7	0.92	0.73	0.56	0.92 (ε_ele_ = ε_pb_ = 1)	0.93	0.93	0.94	0.94 (ε_ele_ = 2, ε_pb_ = 4)	0	
α-thrombin	7	0.64	0.80	0.86	0.89 (ε_ele_ = ε_pb_ = 7)	0.32	0.14	0.75	0.94 (ε_ele_ = 4, ε_pb_ = 6)	2	
cytochrome C peroxidase	18	0.30	0.04	–0.16	0.30 (ε_ele_ = ε_pb_ = 1)	0.50	0.51	0.28	0.51 (ε_ele_ = 1, ε_pb_ = 3)	2	
neuraminidase	8	–0.39	0.00	0.64	0.84 (ε_ele_ = ε_pb_ = 10)	–0.40	–0.39	–0.27	0.83 (ε_ele_ = 9, ε_pb_ = 10)	11	
P450cam	9	0.59	0.64	0.65	0.65 (ε_ele_ = ε_pb_ = 4)	0.67	0.66	0.67	0.67 (ε_ele_ = 2, ε_pb_ = 4)	1	
penicillopepsin	7	–0.12	0.13	0.36	0.48 (ε_ele_ = ε_pb_ = 10)	0.74	0.82	0.70	0.84 (ε_ele_ = 1, ε_pb_ = 8)	4	
DHFR	6	0.19	0.47	0.54	0.54 (ε_ele_ = ε_pb_ = 4)	–0.45	–0.45	–0.40	0.73 (ε_ele_ = 6, ε_pb_ = 5)	1	(81)
COMT	3	–0.95	–0.70	0.40	0.82 (ε_ele_ = ε_pb_ = 10)	0.95	0.95	1.00	1.00 (ε_ele_ = 2, ε_pb_ = 4)	6	
STR	6	–0.85	–0.23	0.29	0.44 (ε_ele_ = ε_pb_ = 10)	0.38	0.48	0.44	0.5 (ε_ele_ = 1, ε_pb_ = 6)	1	

In almost all of the scenarios, using the two-valued ε can be superior to the single-valued one. In avidin complexes, there are no charged amino acids (acidic or basic) in the ligand binding site (within 4 Å). Thus, single-valued ε is quite successful even at the value of 1. Increasing ε indeed worsens the correlation coefficient. On the other hand, the two-valued ε approach yields almost the same performance for avidin complexes. For α-thrombin complexes in which there are only two charged (acidic or basic) amino acids in the ligand binding pocket (by 4 Å), the single-valued ε can get a correlation coefficient from 0.64 to 0.86 by changing the ε value from 1 to 4. The best correlation of 0.89 is observed at ε = 7. The two-valued approach produced a 0.94 correlation with ε_ele_ = 4 and ε_pb_ = 6. For cytochrome C peroxidase, a single-valued dielectric constant could produce the best correlation as only 0.30 for the ε = 1, whereas the correlation has been drastically improved to 0.51 upon defining ε_ele_ = 1 and ε_pb_ = 2. On the other hand, in the case of neuraminidase, in which the binding pocket is highly charged (i.e., 11 amino acids within 4 Å around the ligand), both approaches failed for the low values of ε. In addition, all of the inhibitors of this protein are also −1 charged. Therefore, both approaches give a high correlation when ε is increased up to 10 due to a highly charged protein and charged ligand electrostatic interaction. Even in this case, the two-valued approach can be comparable to the single-valued one. The case of P450cam was similar to that of avidin with relatively lower correlations. The best correlation was 0.65 with the single-valued approach for which ε_ele_ = ε_pb_ = 4, while it was 0.67 with the two-valued approach for which ε_ele_ = 2 and ε_pb_ = 4. The superiority of the two-valued approach was more obvious in the case of penicillopepsin with four charged residues in the binding site. The dielectric constant has been improved from −0.12 to 0.48 by changing the ε value from 1 to 10 in the single-valued approach. On the other hand, using ε_ele_ = 1 and ε_pb_ = 4, we observed a 0.82 correlation coefficient for this enzyme. In the case of DHFR, the two-valued approach produces a high correlation (*R* = 0.73) when ε_ele_ > ε_pb_ (6,5, respectively). Finally, in the case of STR, the ε value must be very high (ε_ele_ = ε_pb_ = 10) to get a fair correlation (*R* = 0.44), whereas the two-valued approach produces better performance (*R* = 0.48) with more descent dielectric constant values (ε_ele_ = 1, ε_pb_ = 4).

## Summary and Conclusions

We have thoroughly revisited single-trajectory approach MMPBSA and MMPBSA_E calculations for selected 54 protein–ligand complex systems, of which experimental values are determined with high accuracy. We have tested predefined coefficients in the nonpolar solvation term as well as our own fitting parameters. Our results have shown that the SPT model is superior to other regimes. We have also investigated the success of ASA/PCAV over SASA/SAV and shown a slight improvement in the SPT regime. In addition, we have implemented ANI-ML potentials in the MM terms of the MMPBSA calculations, which lead to significant improvements over the single- and two-term approaches and somewhat similar to the SPT model. Finally, we have also investigated the use of a two-valued dielectric constant and shown superiority over the single-valued one.

In summary, we for the first time report the success of incorporating ANI-ML potentials in MMPBSA calculations, which could be used in screening large databases in drug discovery more accurately with less computational costs. The ANI implementation can be applied to any uncharged ligand systems. We should note that they have certain limitations in the existence of charged ligands. In addition, using the SPT model rather than a single-term approach for the nonpolar contribution to the solvation free energies can improve the accuracy of the BFE calculations. Finally, modeling the interaction between protein–ligand and solute–solvent by the use of two separate dielectric constant values produces more accurate results than the single-valued definition of both couples.

## Supporting Information Available

Raw data of all outputs is provided as Raw_Results (XLSX)
Supplementary tables of properties of proteins and ligands, MMPBSA, and ANI-PBSA calculations of data set B (PDF)

(XLSX)
(PDF)
