# Supplementary material for: Revisiting MMPBSA by Adoption of MC-Based Surface Area/Volume, ANI-ML Potentials, and Two-Valued Interior Dielectric Constant
Source: J Phys Chem B. 2023 May 12;127(20):4415–29. doi: 10.1021/acs.jpcb.3c00834 (PMC10226125; doi:10.1021/acs.jpcb.3c00834)
Supplement: Supplementary file 2 — jp3c00834_si_002.pdf [file jp3c00834_si_002.pdf]

# Revisiting MM-PBSA by Adoption of MC Based Surface Area/Volume, ANI-ML Potentials and Two Valued Interior Dielectric Constant

*Ebru Akkus[1], Omer Tayfuroglu[1], Muslum Yildiz[2], and Abdulkadir Kocak[1,*]*

* Corresponding to: Abdulkadir Kocak, Email: kocak@gtu.edu.tr, Phone: +902626053083

[1] Department of Chemistry, Gebze Technical University, 41400, Kocaeli/Turkey

[2] Department of Molecular Biology and Genetics, Gebze Technical University, 41400, Kocaeli/Turkey

**Table S1.** Proteins, ligands and their lipophilicity/hydrophilicity values calculated by SwissAdme web server.

| Protein | | | Ligand | | | | | |
|---|---|---|---|---|---|---|---|---|
| Family | ID | Charge | IC50/Ki/Kd (nM) | XLOG P3 | ESOL Log S | ESOL Class | Smile | Ref |
| JNK-1 | 18624 | -3 | 570 | 1.51 | -2.45 | Soluble | CCOC1=N[C@@H](C=C([C@@H]1C#N)N)NC(=O)Cc1ccccc1 | https://doi.org/10.1039/D1SC03472C |
| JNK-1 | 18629 | -3 | 2700 | 2.58 | -3.38 | Soluble | CCOc1c(c(cc(n1)NC(=O)Cc1cc(C)ccc1)N)C#N | https://doi.org/10.1039/D1SC03472C |
| JNK-1 | 18630 | -3 | 190 | 2.58 | -3.38 | Soluble | CCOc1c(c(cc(n1)NC(=O)Cc1ccc(C)cc1)N)C#N | https://doi.org/10.1039/D1SC03472C |
| JNK-1 | 18631 | -3 | 120 | 1.48 | -2.53 | Soluble | CCOC1=N[C@@H](C=C([C@H]1C#N)N)NC(=O)Cc1c(OC)cccc1 | https://doi.org/10.1039/D1SC03472C |
| JNK-1 | 18632 | -3 | 210 | 2.19 | -3.15 | Soluble | CCOc1c(c(cc(n1)NC(=O)Cc1cc(OC)ccc1)N)C#N | https://doi.org/10.1039/D1SC03472C |
| JNK-1 | 18633 | -3 | 180 | 2.19 | -3.15 | Soluble | CCOc1c(c(cc(n1)NC(=O)Cc1ccc(OC)cc1)N)C#N | https://doi.org/10.1039/D1SC03472C |
| JNK-1 | 18634 | -3 | 45 | 2.16 | -3.22 | Soluble | CCOc1c(c(cc(n1)NC(=O)Cc1cc(ccc1OC)OC)N)C#N | https://doi.org/10.1039/D1SC03472C |
| JNK-1 | 18635 | -3 | 4400 | 2.95 | -3.68 | Soluble | CCOc1c(c(cc(n1)NC(=O)Cc1cc(ccc1C)C)N)C#N | https://doi.org/10.1039/D1SC03472C |
| JNK-1 | 18637 | -3 | 35 | 1.34 | -2.88 | Soluble | CCOc1c(c(cc(n1)NC(=O)Cc1cc(c(cc1OC)NC(=O)C)OC)N)C#N | https://doi.org/10.1039/D1SC03472C |
| JNK-1 | 18638 | -3 | 38 | 0.69 | -2.53 | Soluble | CCOC1=N[C@@H](C=C([C@@H]1C#N)N)NC(=O)Cc1cc(c(cc1OC)S(=O)(=O)C)OC | https://doi.org/10.1039/D1SC03472C |
| JNK-1 | 18639 | -3 | 69 | 1.29 | -2.71 | Soluble | N1=C(OCC)C(=C(N)C[C@@H]1NC(=O)Cc1cc(c(cc1OC)N(=O)=O)OC)C#N | https://doi.org/10.1039/D1SC03472C |
| JNK-1 | 18652 | -3 | 14 | 1.83 | -3.47 | Soluble | CC(C)Oc1c(c(cc(n1)NC(=O)Cc1cc(c(cc1OC)S(=O)(=O)C)OC)N)C#N | https://doi.org/10.1039/D1SC03472C |
| JNK-1 | 18658 | -3 | 74 | 1.11 | -2.58 | Soluble | OCCOc1c(c(cc(n1)NC(=O)Cc1cc(ccc1OC)OC)N)C#N | https://doi.org/10.1039/D1SC03472C |
| JNK-1 | 18659 | -3 | 110 | 1.65 | -2.93 | Soluble | COCCOc1c(c(cc(n1)NC(=O)Cc1cc(ccc1OC)OC)N)C#N | https://doi.org/10.1039/D1SC03472C |
| HIV-1 | 2A4F | -4 | 0.06 | 4.36 | -6.15 | Poorly soluble | Oc1ccc(cc1)CCC(=O)N1C[C@@H](O)[C@@H](Cc2ccccc2)N(Cc2cc(OC)c(cc2)O)C(=O)N1Cc1cc(c(cc1)O)OC | https://doi.org/10.1016/j.bmcl.2005.08.093 |
| SARS-CoV | 2GZ7 | 3 | 300 | 4.88 | -5.75 | Moderately soluble | Clc1cc(Cl)c(C)cc1S(=O)(=O)c1c(N(=O)=O)cc(C(F)(F)F)cc1N(=O)=O | https://doi.org/10.1021/jm060207o |
| SARS-CoV | 2GZ8 | 3 | 3000 | 3.99 | -4.69 | Moderately soluble | c1ccccc1C#Cc1ccc(o1)C(=O)S[C@@H]1NN[C@H](N1)C(F)(F)F | https://doi.org/10.1021/jm060207o |
| HIV-1 | 3R0W | -6 | 30.30 | 2.46 | -4.12 | Moderately soluble | CC(C)C[C@@H](C(=O)N)NC(=O)[C@H](CCl)NC(=O)C[C@H](O)[C@H](Cc1ccccc1)NC(=O)[C@@H](C(C)C)NC(=O)c1ccccn1 | https://doi.org/10.1016/j.bbrc.2012.03.096 |
| HIV-1 | 3R0Y | -6 | 4.53 | 3.28 | -4.66 | Moderately soluble | c1nc(ccc1)C(=O)N[C@@H](C(C)C)C(=O)N[C@@H](Cc1ccccc1)[C@@H](O)CC(=O)N[C@@H](C(=O)NCC#C)Cc1ccccc1 | https://doi.org/10.1016/j.bbrc.2012.03.097 |
| HIV-1 | 3SPK | -8 | 3.02 | 6.97 | -7.49 | Poorly soluble | FC(F)(F)c1ccc(nc1)S(=O)(=O)Nc1cc(ccc1)[C@@H](CC)C1=C(C[C@@](OC1=O)(CCC)CCc1ccccc1)O | https://doi.org/10.1016/j.bbrc.2011.08.045 |
| SARS-CoV | 3V3M | 3 | 4800 | 4.97 | -5.46 | Moderately soluble | CC(C)(C)c1ccc(cc1)N(C(=O)c1ccco1)[C@@H](C(=O)NC(C)(C)C)c1cccnc1 | https://doi.org/10.1021/jm301580n |
| HIV-1 | 4KB9 | -6 | 1.90 | 4.76 | -5.95 | Moderately soluble | c1cc(OC)ccc1S(=O)(=O)N(CC(C)C)C[C@@H](O)[C@H](Cc1ccccc1)NC(=O)O[C@H]1CCC[C@@H]2[C@H]1[C@@H]1[C@H](O2)OCC1 | https://doi.org/10.1021/jm400768f |
| SARS-CoV | 4MDS | 3 | 6200 | 2.81 | -4.4 | Moderately soluble | CC(=O)Nc1ccc(cc1)N([C@@H](C(=O)NC(C)(CC)C)c1cccn1C)C(=O)Cn1nnc2c1cccc2 | https://doi.org/10.1016/j.bmcl.2013.08.112 |
| MERS-CoV | 4YOJ | 4 | 410 | 4.31 | -5.43 | Moderately soluble | c1cc(ccc1)C(=O)Nc1ccc(cc1)N(Cc1cscc1)C(=O)Cn1c2ccccc2nn1 | https://doi.org/10.1016/j.bmc.2015.06.039 |
| HIV-1 | 5DGW | -6 | 0.20 | 3.59 | -5.09 | Moderately soluble | CCN[C@@H]1[C@@H]2[C@H](OC1)OCC[C@@H]2OC(=O)N[C@@H](Cc1ccccc1)[C@H](O)CN(CC(C)C)S(=O)(=O)c1ccc(OC)cc1 | https://doi.org/10.1039/C5OB01930C |
| HIV-1 | 5IVS | -4 | 6.00 | 5.45 | -6.49 | Poorly soluble | c1cc(ccc1)CNC(=O)OC[C@H]1NC[C@H](OC1)CCc1ccccc1NC(=O)[C@@H](NC(=O)OC)C(c1ccccc1)c1ccccc1 | https://doi.org/10.1021/acsmedchemlett.6b00135 |
| HIV-1 | 5IVT | -6 | 0.80 | 4.27 | -6.01 | Poorly soluble | c1cc(Cl)ccc1[C@@H](c1cc(cc(c1)F)F)[C@H](N)C(=O)Nc1cncc(F)c1CC[C@H]1OC[C@H](NC1)COC(=O)NCC(F)(F)F | https://doi.org/10.1021/acsmedchemlett.6b00136 |
| HIV-1 | 5TYS | -4 | 0.01 | 4.99 | -6.59 | Poorly soluble | C1CC1N[C@H]1Sc2cc(ccc2N1)S(=O)(=O)N(CC(C)C)C[C@@H](O)[C@H](Cc1cc(cc(c1)F)F)NC(=O)O[C@@H]1[C@@H]2CO[C@H](O)[C@@H]2C[C@@H]1C | https://doi.org/10.7554/eLife.28020.001 |
| HIV-1 | 5ULT | -6 | 2.70 | 3.88 | -5.23 | Moderately soluble | COc1ccc(cc1)S(=O)(=O)N(CC(C)C)C[C@@H](O)[C@H](Cc1ccccc1)NC(=O)O[C@H]1[C@H]2CO[C@@H]3[C@H](C2)[C@H]1CO3 | https://doi.org/10.1021/acs.jmedchem.7b00172 |
| HIV-1 | 5UOV | -6 | 69.00 | 5.69 | -7.09 | Poorly soluble | COc1ccc(cc1)S(=O)(=O)N(CC(C)C)C[C@@H](O)[C@H](Cc1ccccc1)NC(=O)c1cc(ccc1)C(=O)N1CCC[C@@H]1c1nc(C)cs1 | https://doi.org/10.1016/j.bmc.2017.04.005 |

| | | | | | | | | |
|---|---|---|---|---|---|---|---|---|
| HIV-1 | 6CDJ | -6 | 0.03 | 5.8 | -7.2 | Poorly soluble | C1CC1Nc1nc2c(s1)cc(cc2)S(=O)(=O)N(CC(C)C)C[C@@H](O)[C@H](Cc1cc(cc(c1)F)F)NC(=O)O[C@H]1[C@H]2CCO[C@@H]3[C@H]2[C@@H](CO3)C1 | https://doi.org/10.1021/acs.jmedchem.8b00298 |
| HIV-1 | 6CDL | -6 | 0.02 | 5.8 | -7.2 | Poorly soluble | C1CC1Nc1nc2c(s1)cc(cc2)S(=O)(=O)N(CC(C)C)C[C@@H](O)[C@H](Cc1cc(cc(c1)F)F)NC(=O)O[C@@H]1[C@@H]2CCO[C@H]3[C@@H]2[C@H](CO3)C1 | https://doi.org/10.1021/acs.jmedchem.8b00299 |
| HIV-1 | 6VOE | -6 | 0.30 | 4.31 | -5.89 | Moderately soluble | CC(C)Nc1nc2c(o1)cc(cc2)S(=O)(=O)N(CC(C)C)C[C@@H](O)[C@H](Cc1ccccc1)N[C@H](O)O[C@H]1C[C@@H]2[C@@H]3C[C@H]1O[C@@H]3OC2 | https://doi.org/10.1021/acs.jmedchem.0c00202 |
| SARS-CoV-2 | 7L10 | 4 | 4020 | 4.66 | -5.78 | Moderately soluble | c1c(Cl)cc(Cl)cc1c1c(=O)n(cc(c1)c1c(cccc1)C#N)c1cccnc1 | https://doi.org/10.1021/acscentsci.1c00039 |
| SARS-CoV-2 | 7L11 | 4 | 140 | 4.9 | -5.83 | Moderately soluble | CCCOc1cc(cc(c1)Cl)c1cc(cn(c1=O)c1cnccc1)c1c(C#N)cccc1 | https://doi.org/10.1021/acscentsci.1c00040 |
| SARS-CoV-2 | 7L12 | 4 | 128 | 2.9 | -4.98 | Moderately soluble | c1cc(ccc1)COc1cc(cc(c1)Cl)c1cc(cn(c1=O)c1cnccc1)c1c[nH]c(=O)[nH]c1=O | https://doi.org/10.1021/acscentsci.1c00041 |
| SARS-CoV-2 | 7L13 | 4 | 18 | 3.52 | -5.57 | Moderately soluble | c1c(Cl)c(ccc1)COc1cc(cc(c1)Cl)c1cc(cn(c1=O)c1cnccc1)c1c(=O)[nH]c(=O)[nH]c1 | https://doi.org/10.1021/acscentsci.1c00042 |
| SARS-CoV-2 | 7L14 | 4 | 170 | 4.75 | -5.79 | Moderately soluble | c1c(C#N)c(ccc1)c1cc(c(=O)n(c1)c1cnccc1)c1cc(cc(c1)Cl)OCC1CC1 | https://doi.org/10.1021/acscentsci.1c00043 |
| SARS-CoV-2 | 7LMF | 4 | 63 | 3.01 | -4.35 | Moderately soluble | c1nc(c[nH]1)c1ccc(cc1)N(C[C@H]1CSC=C1)C(=O)Cn1c2ccccc2nn1 | https://doi.org/10.1021/acs.jmedchem.1c00598 |
| SARS-CoV | 7LMG | 3 | 148 | 3.28 | -4.63 | Moderately soluble | c1nc(c[nH]1)c1ccc(cc1)N(Cc1cscc1)C(=O)Cn1c2ccccc2nn1 | https://doi.org/10.1021/acs.jmedchem.1c00599 |
| SARS-CoV | 7LMH | 3 | 930 | 4.03 | -5.18 | Moderately soluble | c1cc2c(cc1)n(nn2)CC(=O)N(Cc1cscc1)c1ccc(cc1)c1cccnc1 | https://doi.org/10.1021/acs.jmedchem.1c00600 |
| SARS-CoV | 7LMI | 3 | 109 | 2.45 | -4 | Soluble | c1cc2c(cc1)n(nn2)CC(=O)N(CC1=CCSC1)c1ccc(cc1)c1c[nH]nc1 | https://doi.org/10.1021/acs.jmedchem.1c00601 |
| SARS-CoV-2 | 7M8M | 4 | 120 | 2.3 | -4.24 | Moderately soluble | CCCOc1cc(cc(c1)Cl)c1cc(cn(c1=O)c1cccnc1)c1c(=O)[nH]c(=O)[nH]c1 | https://doi.org/10.1016/j.str.2021.06.002 |
| SARS-CoV-2 | 7M8N | 4 | 100 | 3.26 | -5.28 | Moderately soluble | Cc1ccccc1COc1cc(cc(c1)c1cc(cn(c1=O)c1cccnc1)c1c(=O)[nH]c(=O)[nH]c1)Cl | https://doi.org/10.1016/j.str.2021.06.003 |
| SARS-CoV-2 | 7M8O | 4 | 37 | 3 | -5.14 | Moderately soluble | O=c1[nH]c(=O)[nH]cc1c1cc(c(=O)n(c1)c1cnccc1)c1cc(cc(c1)Cl)OCc1cc(ccc1)F | https://doi.org/10.1016/j.str.2021.06.004 |
| SARS-CoV-2 | 7M8P | 4 | 20 | 3.63 | -5.73 | Moderately soluble | Clc1ccccc1COc1c(c(cc(c1)c1cc(cn(c1=O)c1cccnc1)c1c(=O)[nH]c(=O)[nH]c1)Cl)F | https://doi.org/10.1016/j.str.2021.06.005 |
| SARS-CoV-2 | 7M8X | 4 | 470 | 4.04 | -5.18 | Moderately soluble | COCCOc1cc(cc(c1)C1=C[C@@H](CN(C1=O)c1cnccc1)c1c(C#N)cccc1)Cl | https://doi.org/10.1016/j.str.2021.06.006 |
| SARS-CoV-2 | 7M8Y | 4 | 110 | 3.08 | -4.99 | Moderately soluble | c1cc(ccc1)CCOc1cc(cc(c1)Cl)[C@H]1C=C(CN(C1=O)c1cccnc1)c1c(=O)[nH]c(=O)[nH]c1 | https://doi.org/10.1016/j.str.2021.06.007 |
| SARS-CoV-2 | 7M8Z | 4 | 250 | 1.69 | -4.02 | Moderately soluble | CC(C)(O)CCOc1cc(cc(c1)Cl)c1cc(cn(c1=O)c1cccnc1)c1c(=O)[nH]c(=O)[nH]c1 | https://doi.org/10.1016/j.str.2021.06.008 |
| SARS-CoV-2 | 7M90 | 4 | 250 | 0.56 | -3.51 | Soluble | C1C(=O)NCCN1CCOc1cc(cc(c1)Cl)c1cc(cn(c1=O)c1cnccc1)c1c[nH]c(=O)[nH]c1=O | https://doi.org/10.1016/j.str.2021.06.009 |
| SARS-CoV-2 | 7M91 | 4 | 25 | 3.05 | -4.82 | Moderately soluble | c1ccncc1N1C(=O)C(=C[C@H](C1)c1c(=O)[nH]c(=O)[nH]c1)c1cc(cc(c1)OCCC(F)(F)F)Cl | https://doi.org/10.1016/j.str.2021.06.010 |
| HIV-1 | 7MYP | -4 | 2.40 | 3.15 | -4.73 | Moderately soluble | c1cc(OC)ccc1S(=O)(=O)N(CC(C)C)C[C@@H](O)[C@H](Cc1ccccc1)NC(=O)O[C@@H]1[C@H]2[C@@H](OC1)OC[C@@H]2OC | https://doi.org/10.1016/j.bbrc.2021.05.094 |
| SARS-CoV-2 | 7N44 | 4 | 42 | 2.36 | -4.75 | Moderately soluble | Cc1scnc1COc1cc(cc(c1)Cl)c1cc(cn(c1=O)c1cccnc1)c1c(=O)[nH]c(=O)[nH]c1 | https://doi.org/10.1021/acsmedchemlett.1c00326 |
| SARS-CoV-2 | 7P51 | 4 | 54000 | 2.1 | -3.19 | Soluble | c1cc(Cl)cnc1NC(=O)[C@@H]1CC(=O)c2c1cccc2 | https://doi.org/10.1002/anie.202109965 |
| SARS-CoV-2 | 7TE0 | 4 | 3.11 | 1.47 | -3.1 | Soluble | C1CNC(=O)[C@@H]1C[C@@H](CN)NC(=O)[C@@H]1[C@H]2C(C)(C)[C@H]2CN1C(=O)[C@H](C(C)(C)C)NC(=O)C(F)(F)F | https://doi.org/10.1021/acs.jmedchem.2c00404 |
| Urokinase | 1owh | -3 | 40 | 0.91 | -2.57 | Soluble | [nH2][c]([nH2])c1cc2ccc(cc2cc1)C(=O)Nc1ccc(cc1)C[NH3+] | http://dx.doi.org/10.1021/jm0300072 |
| Urokinase | 1sqa | -3 | 0.62 | 1.07 | -3.15 | Soluble | [NH3+]Cc1ccc(NC(=O)c2cc(Nc3ncccn3)c3cc(ccc3c2)[c]([nH2])[nH2])cc1 | http://dx.doi.org/10.1016/j.bmcl.2004.04.030 |

| | | | | | | | | |
|---|---|---|---|---|---|---|---|---|
| HSP82 | 2fxs | 9 | 870 | 2.57 | -3.8 | Soluble | COC(=O)c1c(O)cc(O)c(Cl)c1CCC(=O)Nc1cc(O)c(OC)cc1O | http://dx.doi.org/10.1016/j.jmb.2009.03.071 |
| HSP90 | 2iwx | 9 | 210 | 4.48 | -5.03 | Moderately soluble | C1(=O)c2c(cc(c(c2CC(=O)CCCCC/C=C/CCO1)Cl)O)O | http://dx.doi.org/10.1016/j.chembiol.2006.09.015 |
| Endothiapepsin | 2v00 | 14 | 220000 | 0.93 | -2.12 | Soluble | N1C(=NC(=CC1=O)CCc1ccccc1)N | http://dx.doi.org/10.1021/jm070825k |
| HSP90 | 2vw5 | 9 | 3 | 4.06 | -5.44 | Moderately soluble | O=C1/C(=C/CC[C@H](C)[C@H](/C(=C/[C@@H]([C@H]([C@H](C[C@@H](Cc2cc(cc(N1)c2)O)C)OC)O)C)/C)OC(=O)N)/C | http://dx.doi.org/10.1021/jm8006068 |
| HSP90 | 2yki | 7 | 0.35 | 3.64 | -5.27 | Moderately soluble | c1ncc2NC(=Nc2c1)c1cccc2[C@H](NC(=O)c3ccnc4NC=Cc34)c3c(-c12)cccc3 | http://dx.doi.org/10.1021/jm200784m |
| HSP90 | 3b27 | 10 | 6900 | 2.38 | -3.33 | Soluble | CSc1nc(nc(n1)N)c1ccccc1Cl | http://dx.doi.org/10.1016/j.bmcl.2011.08.001 |
| AR | 3b5r | -5 | 1.7 | 3.67 | -4.59 | Moderately soluble | c1c(F)c(Cl)ccc1OC[C@@](O)(C)C(=O)Nc1cc(c(cc1)C#N)C(F)(F)F | http://dx.doi.org/10.1016/j.bmcl.2008.09.002 |
| AR | 3b68 | -5 | 4 | 3.18 | -4.21 | Moderately soluble | CC(=O)Nc1ccc(cc1)OC[C@@](O)(C)C(=O)Nc1cc(c(cc1)N(=O)=O)C(F)(F)F | http://dx.doi.org/10.1016/j.bmcl.2008.09.002 |
| AR | 3g0w | -5 | 0.3 | 3.21 | -4.15 | Moderately soluble | C1N2[C@@H]([C@H](C1)O)[C@@H](O/C/2=N\c1c(c(c(cc1)C#N)Cl)C)C(F)(F)F | http://dx.doi.org/10.1021/jm801583j |
| b-lactamase | 3g2z | -1 | 4400000 | 0.11 | -1.17 | Very soluble | O=C1C=C(CCC1)NC1=NN=NN1 | http://dx.doi.org/10.1038/nchembio.155 |
| b-lactamase | 3gr2 | -3 | 3000000 | 0.16 | -1.28 | Very soluble | O=C1[C@H](CC)C(=NN1C1=NN=NN1)C | http://dx.doi.org/10.1073/pnas.0813029106 |
| Pim kinase | 3jya | 13 | 130.1 | 3.31 | -4.21 | Moderately soluble | C1(=O)NC=NC2=C1Sc1c(Cl)ccc(Cl)c21 | https://doi.org/10.1021/jm900943h |
| BRD4 | 3p5o | -1 | 50.5 | 2.85 | -4.29 | Moderately soluble | Clc1ccc(cc1)C1=N[C@H](C2=NN=C(N2c2c1cc(cc2)OC)C)CC(=O)NCC | http://dx.doi.org/10.1038/nature09589 |
| Endothiapepsin | 3prs | 14 | 15 | 6.05 | -6.99 | Poorly soluble | C1=C(SC=N1)COC(=O)N[C@H]([C@H](C[C@H](Cc1ccccc1)NC(=O)[C@@H](NC(=O)N(CC1=CSC(=N1)C(C)C)C)C(C)C)O)Cc1ccccc1 | http://dx.doi.org/10.1002/cmdc.201100490 |
| HSP90 | 3rlr | 13 | 30 | 4.07 | -4.91 | Moderately soluble | n1c2NC(=C(c2c(c2cc(c(cc2Cl)Cl)OC)nc1C)C#N)C | http://dx.doi.org/10.1016/j.bmcl.2011.04.130 |
| BRD4 | 3u5j | -1 | 2460 | 2.12 | -3.6 | Soluble | c12cc(Cl)ccc1N1C(=NN=C1CN=C2c1ccccc1)C | http://dx.doi.org/10.1016/j.bmc.2011.10.080 |
| Endothiapepsin | 3wz8 | 14 | 1500 | 6.23 | -6.38 | Poorly soluble | O=C(CCNc1ccccc1)NC1=C(C(=CS1)c1ccccc1)C(=O)NCc1ccccc1 | http://dx.doi.org/10.1002/anie.201411206 |
| FXIa | 4cra | -4 | 60 | 2.73 | -4.62 | Moderately soluble | C(=O)(c1ccc2c(c1)C(=CC(=O)N2)O)N[C@H](C(=O)NCc1cccc2c1ccc(n2)N)Cc1ccccc1 | http://dx.doi.org/10.1371/journal.pone.0113705 |
| b-lactamase | 4de1 | -1 | 1100 | 1.68 | -3.17 | Soluble | O=C(c1cc2=CNN=c2cc1)Nc1cc(ccc1)C1=NN=NN1 | http://dx.doi.org/10.1021/jm2014138 |
| PDE10A | 4llx | 5 | 1300000 | 0.67 | -1.52 | Very soluble | Cc1cc(C)nc(N)n1 | https://doi.org/10.1177/1087057113516493 |
| BRD4 | 4lzs | -1 | 16000 | 1.2 | -1.87 | Very soluble | C1(=C(C)NC(=C1CC)C(=O)NC)C(=O)C | https://doi.org/10.1021/acs.jmedchem.5b01267 |
| FXIa | 4x6p | -5 | 5 | 4.16 | -5.86 | Moderately soluble | N1=C([C@@H](NC(=O)/C=C/c2cc(ccc2N2C=NN=N2)Cl)Cc2ccccc2)NC=C1c1cc2c(cc1)C(=NN2)N | https://doi.org/10.1016/j.bmcl.2015.01.028 |

**Table S2.** MMPBSA calculated by one term, two term and SPT approaches for non-polar contribution to solvation free energy tested on dataset B, in which no fitting was performed (instead parameters of dataset A were applied).

| | **SASA_only** | | | **SAV_only** | | | **SAV+Disp** | | **SPT** |
|---|---|---|---|---|---|---|---|---|---|
| $\varepsilon_{int}$=1 | GB1=PB4 γ=0.0072 | GB2=PB5 γ=0.005 | Our γ=0.0477 | GB1=PB4 γ=0.0072 | GB2=PB5 γ=0.005 | Our γ=0.0445 | PB1 γ=0.0378, b=-0.5692 | Our γ=0.1116 | Our γ=0.0492, p=-0.001 |
| **PI** | 0.82 | 0.80 | 0.88 | 0.80 | 0.79 | 0.88 | 0.21 | 0.87 | 0.88 |
| **Pearson R** | 0.81 | 0.79 | 0.88 | 0.80 | 0.79 | 0.87 | 0.14 | 0.85 | 0.87 |
| **Spearman R** | 0.81 | 0.78 | 0.86 | 0.78 | 0.78 | 0.86 | 0.20 | 0.86 | 0.86 |
| **MUE** | 15.82 | 14.27 | 72.11 | 15.76 | 14.30 | 67.66 | 10.02 | 70.98 | 125.15 |
| **MUEtr** | 7.57 | 7.34 | 20.87 | 7.87 | 7.55 | 22.28 | 6.53 | 23.52 | 34.77 |
| **MUEsc** | 1.58 | 1.66 | 1.21 | 1.63 | 1.70 | 1.25 | 15.97 | 1.37 | 1.26 |
| $\varepsilon_{int}$=2.1 | | | γ=0.0266 | | | γ=0.0249 | | γ=0.0919 | γ=0.0248, p=0.001 |
| **PI** | 0.89 | 0.89 | 0.89 | 0.90 | 0.89 | 0.88 | 0.61 | 0.88 | 0.88 |
| **Pearson R** | 0.89 | 0.89 | 0.89 | 0.89 | 0.88 | 0.88 | 0.62 | 0.86 | 0.88 |
| **Spearman R** | 0.87 | 0.88 | 0.86 | 0.88 | 0.88 | 0.86 | 0.59 | 0.87 | 0.85 |
| **MUE** | 25.41 | 23.79 | 62.96 | 25.35 | 23.74 | 59.30 | 3.95 | 62.61 | 63.86 |
| **MUEtr** | 9.23 | 8.85 | 18.59 | 9.47 | 9.01 | 19.16 | 3.97 | 20.10 | 17.92 |
| **MUEsc** | 1.11 | 1.12 | 1.18 | 1.13 | 1.14 | 1.16 | 2.84 | 1.30 | 1.25 |
| **PI: predictive index, MUE: Mean unsigned error; MUEtr: Mean unsigned error after subtraction of average signed error, MUEsc: MUE rescaled by slope and intercept of the predicted vs experimental results** | | | | | | | | | |

**Table S3.** MMPBSA calculated by Zeo++ produced SA terms for non-polar contribution to solvation free energy tested on dataset B, in which no fitting was performed (instead parameters of dataset A were applied).

| | **One-term** | | | **Two-term** | | **SPT** | |
|---|---|---|---|---|---|---|---|
| $\varepsilon_{int}$=1 | ASA only γ=0.046 | PCAV only γ=0.046 | POAV only γ=-0.027 | PCAV+Disp γ=0.115 | POAV+Disp γ=-0.381 | ASA+PCAV γ=0.031, p=-0.019 | ASA+POAV γ=0.045, p=-0.003 |
| **PI** | 0.83 | 0.88 | 0.34 | 0.82 | -0.57 | 0.79 | 0.79 |
| **Pearson R** | 0.85 | 0.85 | 0.13 | 0.78 | -0.50 | 0.78 | 0.79 |
| **Spearman R** | 0.79 | 0.87 | 0.23 | 0.81 | -0.64 | 0.74 | 0.74 |
| **MUE** | 70.74 | 52.24 | 22.73 | 45.17 | 26.01 | 71.67 | 68.70 |
| **MUEtr** | 19.55 | 19.45 | 11.63 | 19.28 | 24.27 | 19.70 | 18.54 |
| **MUEsc** | 1.49 | 1.32 | 17.05 | 1.78 | 3.86 | 1.94 | 1.86 |
| $\varepsilon_{int}$=2.1 | γ=0.025 | γ=0.025 | γ=-0.017 | γ=0.094 | γ=-0.028 | γ=0.017, p=-0.009 | γ=0.024, p=-0.004 |
| **PI** | 0.85 | 0.89 | 0.42 | 0.87 | -0.44 | 0.71 | 0.80 |
| **Pearson R** | 0.86 | 0.88 | 0.40 | 0.81 | -0.44 | 0.74 | 0.79 |
| **Spearman R** | 0.82 | 0.88 | 0.32 | 0.86 | -0.50 | 0.64 | 0.74 |
| **MUE** | 62.08 | 45.74 | 26.40 | 38.67 | 19.65 | 63.50 | 59.61 |
| **MUEtr** | 17.65 | 16.04 | 11.44 | 16.02 | 20.49 | 18.19 | 16.45 |
| **MUEsc** | 1.36 | 1.16 | 5.12 | 1.65 | 4.42 | 2.09 | 1.79 |
| **PI: predictive index, MUE: Mean unsigned error; MUEtr: Mean unsigned error after subtraction of average signed error, MUEsc: MUE rescaled by slope and intercept of the predicted vs experimental results** | | | | | | | |

**Table S4.** ANI_PBSA calculated by Zeo++ produced SA terms for non-polar contribution to solvation free energy tested on dataset B, in which no fitting was performed (instead parameters of dataset A were applied).

| | **One-term** | | | **Two-term** | | **SPT** | |
|---|---|---|---|---|---|---|---|
| $\varepsilon_{int}$=1 | ASA only a=1.4993, γ=0.0662 | PCAV only a=2.4414, γ=-0.0212 | POAVonly a=2.1347, γ=-0.0498 | PCAV+EDisp a=2.9684, γ=0.0072 | POAV+EDisp a=2.922, γ=-0.07088 | ASA_PCAV a=1.6755, γ=0.1139, p=-0.0669 | ASA_POAV a=1.5568, γ=0.0562, p=-0.0300 |
| **PI** | 0.63 | 0.69 | 0.59 | 0.66 | 0.44 | 0.54 | 0.57 |
| **Pearson R** | 0.64 | 0.66 | 0.60 | 0.62 | 0.47 | 0.52 | 0.59 |
| **Spearman R** | 0.56 | 0.65 | 0.52 | 0.64 | 0.36 | 0.44 | 0.48 |
| **MUE** | 50.35 | 30.91 | 33.67 | 25.67 | 22.48 | 50.16 | 47.62 |
| **MUEtr** | 20.05 | 19.28 | 16.86 | 20.29 | 16.73 | 20.85 | 18.99 |
| **MUEsc** | 2.84 | 2.68 | 3.23 | 2.97 | 4.35 | 3.94 | 3.25 |
| $\varepsilon_{int}$=2.1 | a=1.1029, γ=0.0482 | a=1.8698, γ=-0.0232 | a=1.5612, γ=0.0389 | a=2.3969, γ=0.0052, | a=2.3485, γ=-0.0599 | a=1.2596, γ=0.0907, p=-0.0596 | a=1.1504, γ=0.0399, p=-0.0248 |
| **PI** | 0.81 | 0.84 | 0.80 | 0.77 | 0.59 | 0.66 | 0.75 |
| **Pearson R** | 0.78 | 0.78 | 0.75 | 0.72 | 0.58 | 0.64 | 0.74 |
| **Spearman R** | 0.77 | 0.83 | 0.76 | 0.77 | 0.54 | 0.57 | 0.69 |
| **MUE** | 47.34 | 31.39 | 35.17 | 24.97 | 22.94 | 47.17 | 45.09 |
| **MUEtr** | 15.82 | 15.80 | 13.32 | 17.66 | 15.25 | 16.47 | 14.82 |
| **MUEsc** | 1.87 | 1.98 | 2.10 | 2.25 | 3.22 | 2.60 | 2.08 |

**PI: predictive index, MUE: Mean unsigned error; MUEtr: Mean unsigned error after subtraction of average signed error, MUEsc: MUE rescaled by slope and intercept of the predicted vs experimental results**
